# MEK inhibition induces apoptosis in osteosarcoma cells with constitutive ERK1/2 phosphorylation

**DOI:** 10.18632/genesandcancer.91

**Published:** 2015-11

**Authors:** Zuzanna Baranski, Tijmen H. Booij, Marieke L. Kuijjer, Yvonne de Jong, Anne-Marie Cleton-Jansen, Leo S. Price, Bob van de Water, Judith V. M. G. Bovée, Pancras C.W. Hogendoorn, Erik H.J. Danen

**Affiliations:** ^1^ Division of Toxicology, Leiden/Academic Center for Drug Research, Leiden University, Leiden, The Netherlands; ^2^ Department of Pathology, Leiden University Medical Center, Leiden, The Netherlands; ^3^ Department of Biostatistics and Computational Biology, Dana-Farber Cancer Institute, Boston, MA, USA; ^4^ Department of Biostatistics, Harvard T.H. Chan School of Public Health, Boston, MA, USA

**Keywords:** osteosarcoma, MEK, pharmacological inhibition, 3D culture, ERK phosphorylation

## Abstract

Conventional high-grade osteosarcoma is the most common primary bone cancer with relatively high incidence in young people. Recurrent and metastatic tumors are difficult to treat. We performed a kinase inhibitor screen in two osteosarcoma cell lines, which identified MEK1/2 inhibitors. These inhibitors were further validated in a panel of six osteosarcoma cell lines. Western blot analysis was performed to assess ERK activity and efficacy of MEK inhibition. A 3D culture system was used to validate results from 2D monolayer cultures. Gene expression analysis was performed to identify differentially expressed gene signatures in sensitive and resistant cell lines. Activation of the AKT signaling network was explored using Western blot and pharmacological inhibition. In the screen, Trametinib, AZD8330 and TAK-733 decreased cell viability by more than 50%. Validation in six osteosarcoma cell lines identified three cell lines as resistant and three as sensitive to the inhibitors. Western blot analysis of ERK activity revealed that sensitive lines had high constitutive ERK activity. Treatment with the three MEK inhibitors in a 3D culture system validated efficacy in inhibition of osteosarcoma viability. MEK1/2 inhibition represents a candidate treatment strategy for osteosarcomas displaying high MEK activity as determined by ERK phosphorylation status.

## INTRODUCTION

Osteosarcoma is the most common primary malignant bone tumor occurring predominantly in children and adolescents, as well as in people older than 40 years of age. It is thought to arise from mesenchymal stem cells that are capable of producing osteoid [1, 2]. At the moment of diagnosis, 10-20% of the patients present with metastasis. About 30-40% of the patients with localized osteosarcoma will present with relapse mainly as lung metastasis. Patients with recurrence have very poor prognosis with 23-33% 5-year overall survival [3]. Therefore, new effective therapies are urgently needed to improve the prognosis of osteosarcoma patients.

Screening a kinase inhibitor library of pre-clinical or clinically approved drugs provides the possibility of identifying novel candidate treatments for osteosarcoma that can be translated to the clinic.

Receptor tyrosine kinases (RTKs) are frequently hyperactive through mutation or overexpression in cancer causing aberrant activation of downstream signaling cascades, including Ras/Raf/MEK/ERK [4]. This cascade is known to be involved in cell survival, proliferation, and differentiation by regulating the activation of transcription factors such as c-Myc, c-Fos, Ets, and Elk-1 [5, 6]. In osteosarcoma, RTKs such as EGFR [7], KIT [8], FGFR1 [9], and IGFR-1 [10] were found to be amplified or upregulated. Furthermore, ERK pathway activation was reported in 67% of osteosarcomas analyzed [11]. Genetic screens also identified PI3K/AKT signaling as a driver of osteosarcoma [12, 13] and inhibition of this pathway is a potential treatment for osteosarcoma [14]. Inhibition of overexpressed or mutant RTKs is a candidate for cancer treatment but often leads to compensatory activation of another RTK driving cancer cell survival and proliferation [15]. Inhibition of common downstream effector kinases could be an effective way to circumvent such resistance. In this study, we performed a kinase inhibitor screen to identify candidate targets for human osteosarcoma, and identified MEK inhibitors as possible therapeutic targets in cells with constitutive ERK activation.

## RESULTS

### Kinase inhibitor screen for inhibition of osteosarcoma cell viability identifies MEK inhibitors

**Figure 1:**
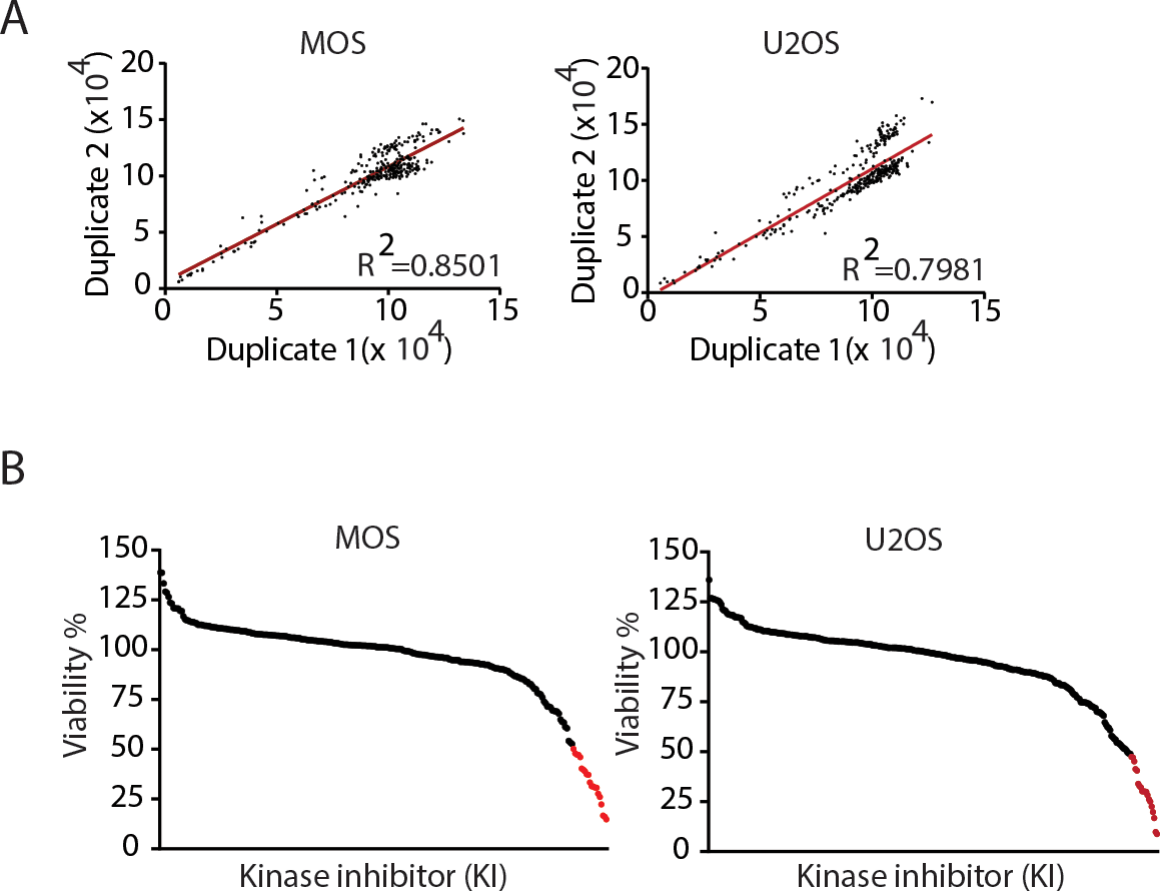
Kinase inhibitor screen in two human osteosarcoma cell lines A) The screen was performed in MOS and U2OS cell lines in duplicate. The graphs represent the goodness of fit of the screens. B) All results were normalized to DMSO and hits are defined by <50% viability (red).

**Figure 2:**
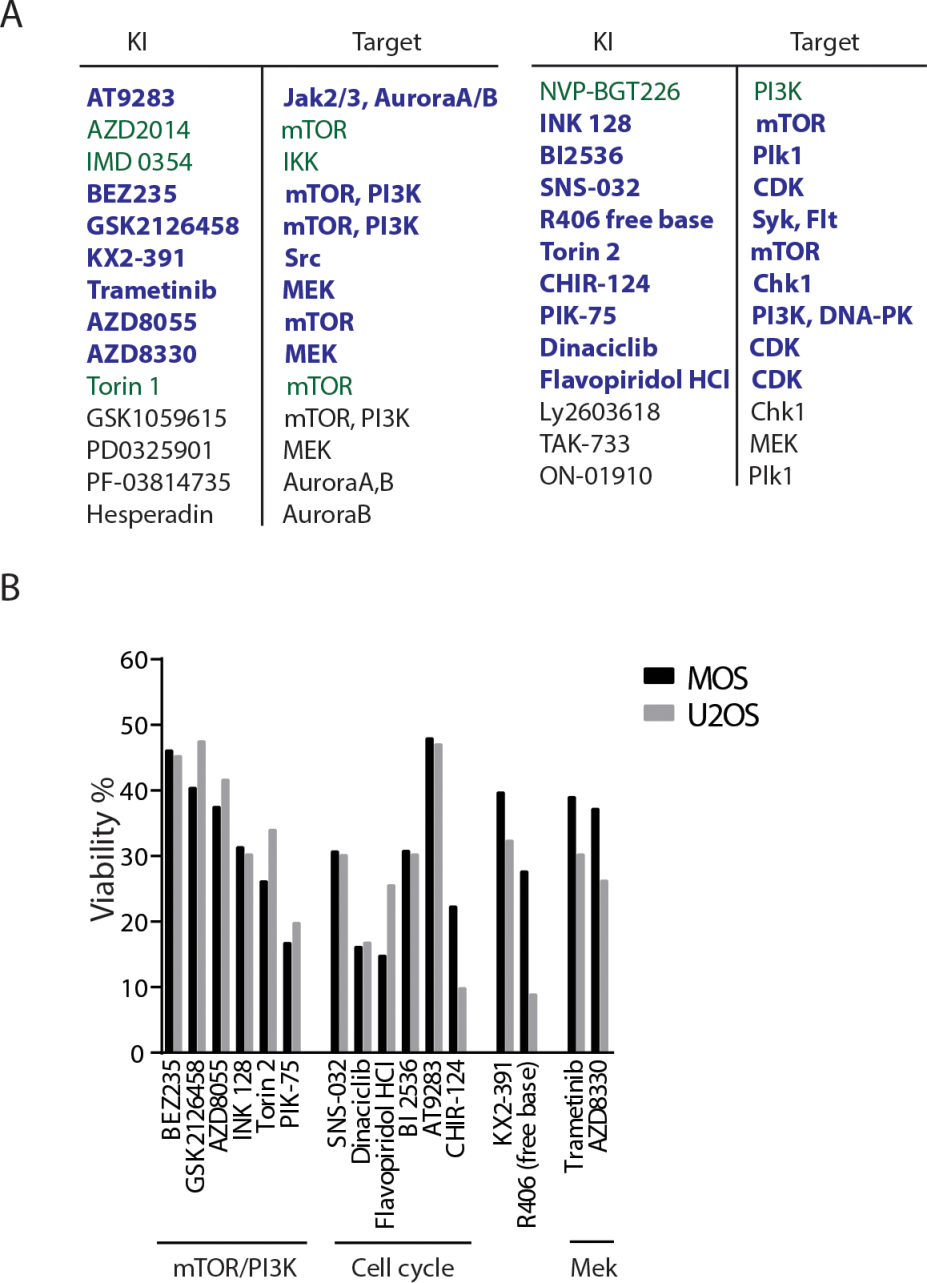
Selection of hits in two human osteosarcoma cell lines A) List of hits common to both cell lines (bold blue), only in MOS (green) and only in U2OS cells (black). B) Bar graphs representing the hits common to both cell lines, their viability score relative to DMSO, and known biological activity.

A library composed of 273 kinase inhibitors was used to screen for inhibitors that, as a single agent, decreased viability of osteosarcoma cells. MOS and U2OS were exposed to a concentration of 1μM for 72 hours and viability was determined by measuring ATP production. Each screen was performed in duplicate with a goodness of fit (R2) of 0.8501 for the screen in MOS and 0.7981 in U2OS (Figure 1A). All values were normalized to DMSO condition, and the candidates that exhibited less than 50% viability were considered a hit (Figure 1B). Under this criterium, we identified 16 inhibitors in common for MOS and U2OS of which, six targeted the PI3K/mTOR pathway (BEZ235, GSK2126458, AZD8055, Torin 2, INK-128, PIK-75), six targeted the cell cycle (AT9283, BI2536, SNS-032, CHIR-124, dinaciclib and flavopiridol HCl), one targted Src (KX-391), one targeted Syk and Flt (R406 free base), and two were MEK1/2 inhibitors (Figure 2A, B). The PI3K/mTOR pathway has been implicated in osteosarcoma cell survival and proliferation *in vivo* [16]. Dinaciclib and flavoripirol were previously reported to induce apoptosis in osteosarcoma cells [17, 18]. Plk1 inhibition has been shown to cause cell death in osteosarcoma cells and its expression correlates with overall survival in osteosarcoma patients [19-21]. Here we focused on three MEK1/2 inhibitors: Trametinib and AZD8330, which were common in MOS and U2OS, and TAK-733, which was a hit in U2OS (in MOS treatment with TAK-733 showed 71% remaining viability).

### MEK1/2 inhibition leads to apoptosis in cells with constitutive ERK activation

**Figure 3:**
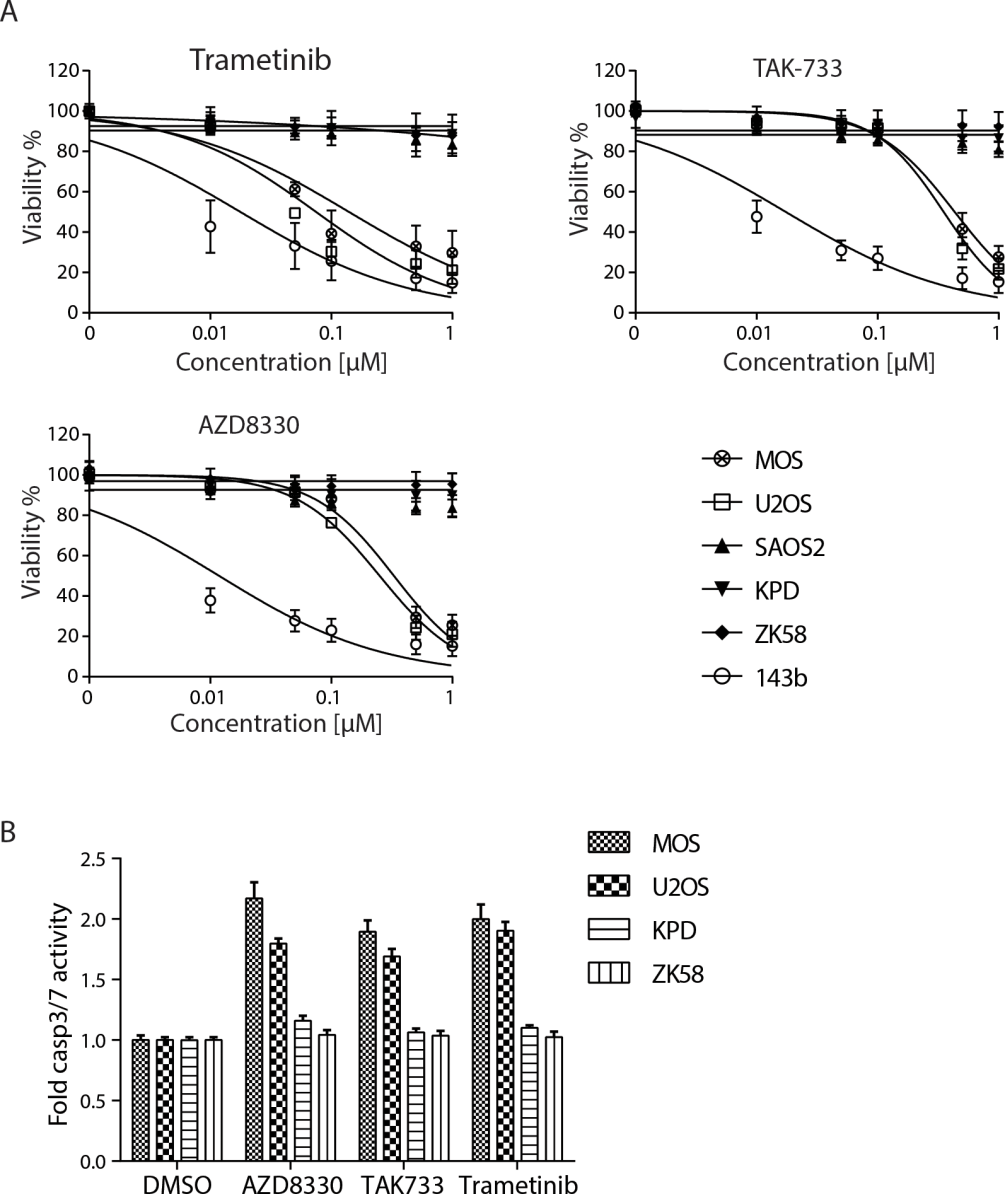
Validation of three MEK inhibitors in 6 osteosarcoma cell lines A) Dose response curves for Trametinib, AZD8330 and TAK-733 in 6 osteosarcoma cell lines as indicated. Cells were exposed for 72 hours. Each graph represents mean±s.e.m. of three replicates. B) Caspase 3/7 activity in presence of indicated inhibitors relative to DMSO in 4 osteosarcoma cell lines. The graph is a representative experiment of 3 independent experiments, each performed in triplicate. Mean±s.d. is shown.

The activity of these three inhibitors was tested using concentration ranges in six osteosarcoma cell lines: MOS, U2OS, KPD, ZK58, 143b and Saos-2 (Figure 3A). All three inhibitors decreased viability of MOS and U2OS and strongly affected 143b. By contrast, viability of KPD, ZK58 and Saos-2 was not affected by any of the three inhibitors. A capase3/7 activity assay confirmed that exposure to 0.5μM of each of the drugs induced apoptosis in MOS and U2OS, but not in KPD and ZK58 cells (Figure 3B).

**Figure 4:**
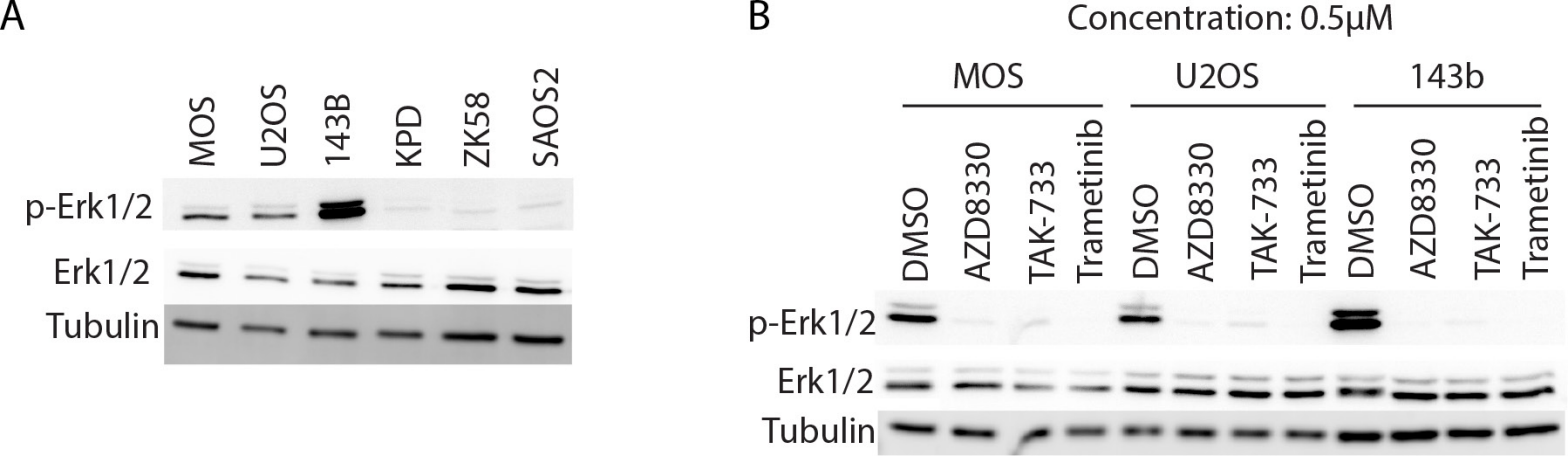
Western blot analysis of ERK phosphorylation in 6 osteosarcoma cell lines and effect of MEK inhibition A) Western blot analysis of total ERK and phospho-ERK in 6 osteosarcoma cell lines. B) Western blot analysis of total ERK and phospho-ERK in MOS, U2OS and 143b osteosarcoma cell lines after 6 hours treatment with DMSO or 0.5μM of the indicated MEK inhibitors.

Next, we asked if the observed differences in the response to MEK inhibition was related to the status of MEK activity, as measured by phosphorylation of the MEK target, ERK. Indeed, 143b, which was the most sensitive cell line, is Ki-*ras*+ transformed [22] and showed the most prominent ERK phosphorylation, followed by the other two sensitive cell lines, MOS and U2OS (Figure 4A). The resistant cell lines KPD, ZK58 and Saos-2 showed no constitutive ERK activation. Exposing MOS, U2OS and 143b to a concentration of 0.5μM of Trametinib, AZD8330 or TAK-733 for 6 hours, led to loss of ERK phosphorylation indicating effective MEK inhibition (Figure 4B).

### Validation of MEK inhibition in a 3D cell culture system

**Figure 5:**
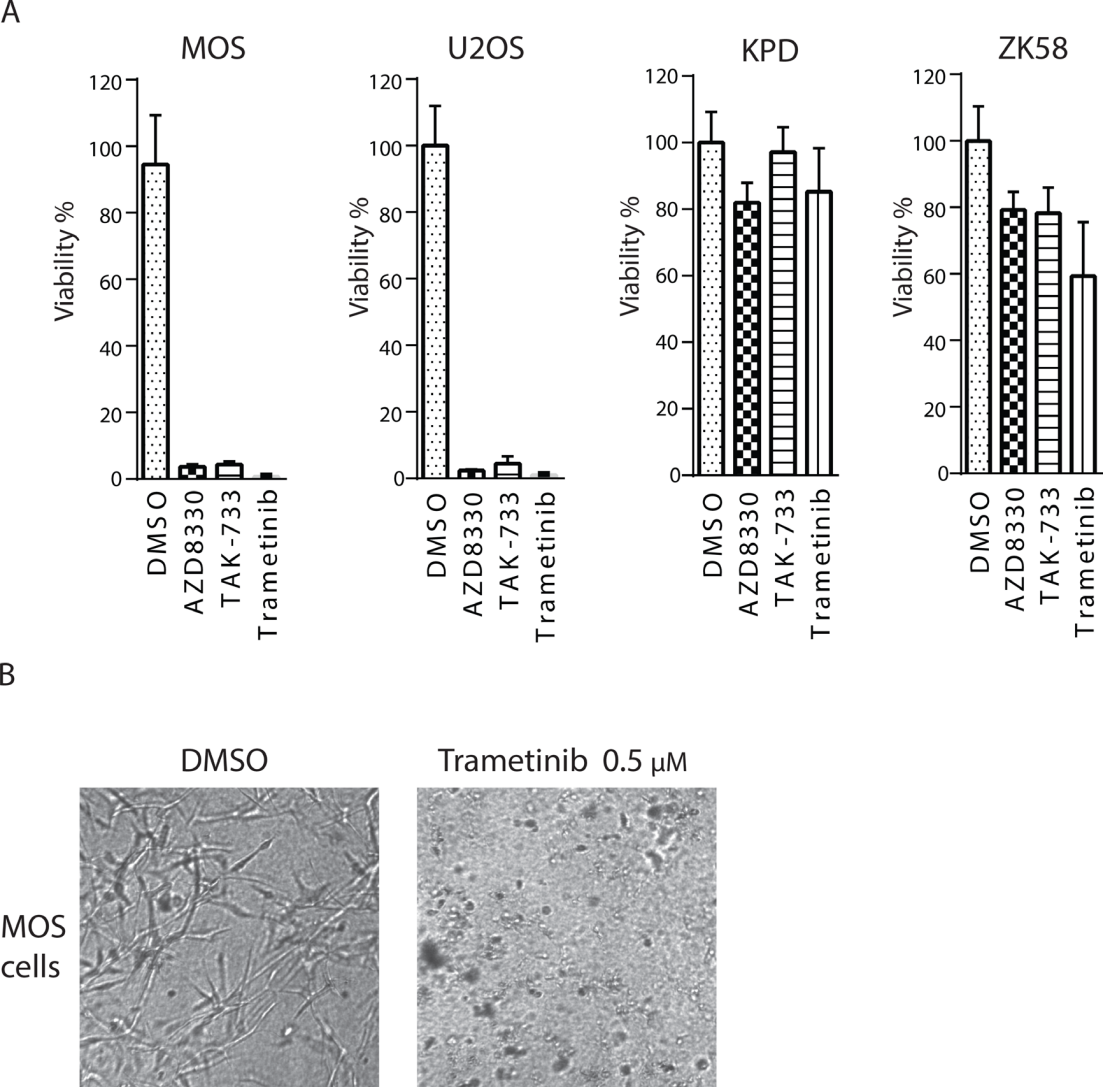
Validation of sensitivity to MEK inhibitors in a 3D culture system A) MOS, U2OS, KPD and ZK58 cells were re-suspended in a collagen-matrigel mix and 3D cultures were subsequently exposed to 0.5μM of the MEK inhibitors for 72 hours. Graphs are a representative experiment of two replicates, each performed in quadruplicate. Mean±s.d is shown. B) Representative images of 3D MOS cultures in absence or presence of Trametinib.

We made use of 3D cultures of identified sensitive and resistant cell lines to further validate the effect of Trametinib, AZD8330 and TAK-733. MOS, U2OS, KPD, and ZK58 were suspended in a collagen-matrigel mixture, and exposed 24 hours later to 0.5μM of each inhibitor for a period of 72 hours. As observed in 2D cultures, MOS and U2OS cells died in the presence of each of the three inhibitors whereas KPD and ZK58 were not affected (Figure 5A, 5B).

### Potential mechanisms of resistance in cell lines not sensitive to MEK inhibition

**Figure 6:**
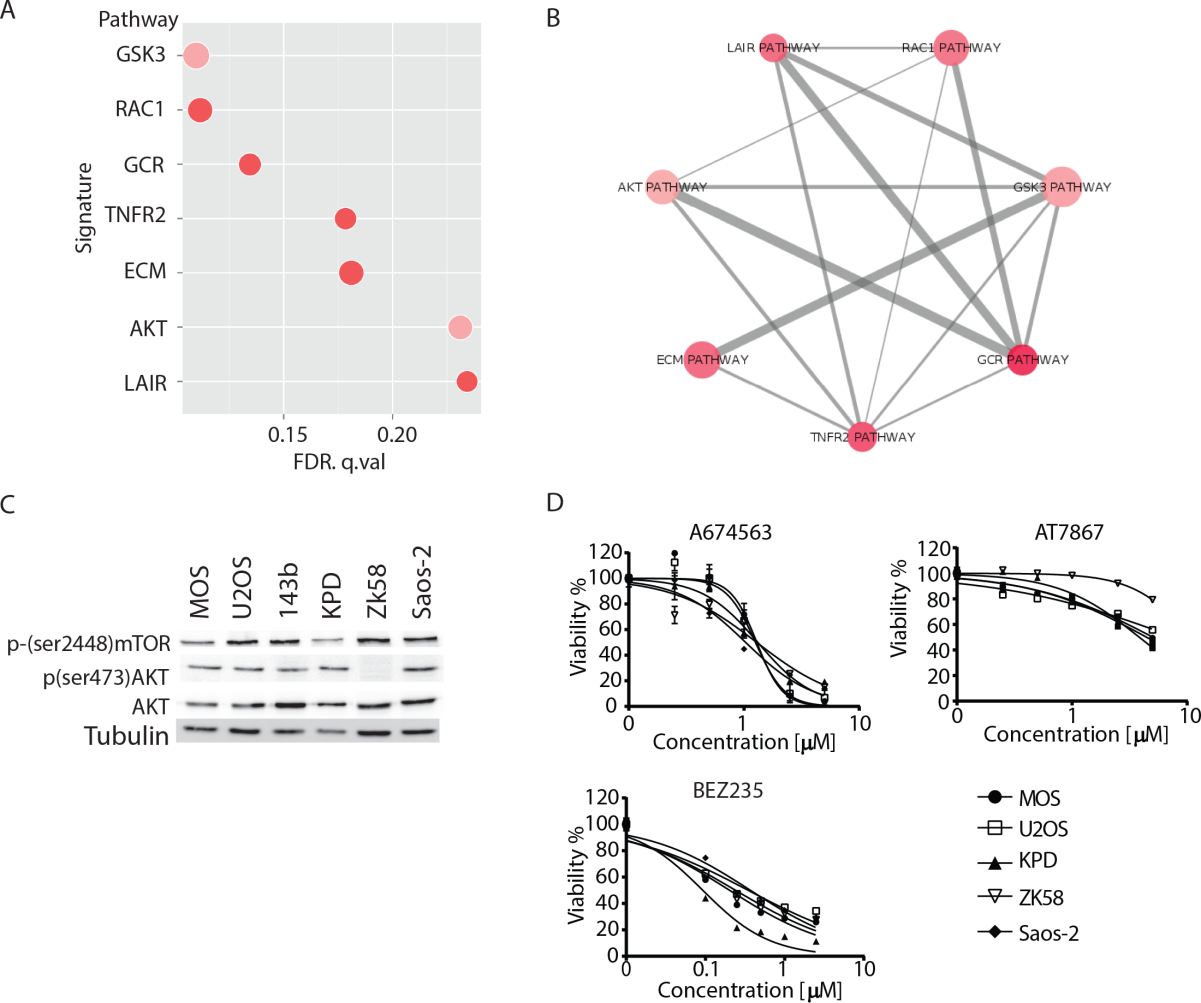
Analysis of AKT pathway and its pharmacological inhibition A) Plot representing the 7 signatures that were significantly enriched (FDR< 0.25) in the cell lines resistant to MEK inhibition based on gene expression data. Pink/red represents the enrichment score (red>pink), and size represents the gene set size of the signature. B) Schematic representation of similarity between the 7 signatures. Pink/red represents the enrichment score (red>pink), and the line width represents the number of genes shared between signatures. C) Western blot analysis of total AKT, phosho(Ser473)-AKT and phospho-(Ser2448)-mTOR in the indicated osteosarcoma cell lines. D) Dose response curves for the indicated AKT-mTOR inhibitors in the indicated osteosarcoma cell lines. Mean±s.d for experiment performed in triplicate is shown.

Our data indicated that MEK1/2 inhibition could be used to treat osteosarcomas that present with constitutive ERK activation but not in cases where MEK activity is low. *Ras/Raf* mutations are strong predictors for sensitivity to MEK inhibition [23, 24] explaining sensitivity of 143b. We searched for mutations in exons or splice sites in the genes *MEK1, MEK2, A-Raf, B-Raf, C-Raf, EGFR, FGFR, IGFR1, K-Ras, H-Ras* and *N-Ras* in all cell lines used, employing a previously published method [25] but could not identify mutations that may explain high constitutive ERK phosphorylation in MOS or U2OS (data not shown). Next, we performed a pathway analysis on gene expression differences in sensitive (MOS, U2OS and 143b) versus resistant (KPD, ZK58 and Saos-2) cell lines [26]. This analysis revealed 7 signatures with enrichment of differentially expressed genes (Figure 6A). One of the signatures was the AKT pathway, which had positive fold change for 15/22 genes upregulated in the resistant cell lines (Figure 6C). However, Western blot analysis of phospho-AKT(Ser473) showed active AKT in all cell lines except ZK58 (Figure 6B). Similarly, mTOR, a downstream target of AKT signaling, was not differentially activated between sensitive and resistant cell lines (Figure 6C). In agreement, all cell lines responded similarly to inhibition of AKT signaling using A674563 (inhibits AKT1 selectively) or AT7867 (inhibits AKT1/2/3) and were highly sensitive to a dual PI3K/mTOR inhibitor, BEZ235 (Figure 6D). These data indicate that other differentially activated signaling pathways, rather than the predicted difference in AKT activity underlie differential sensitivity of the osteosarcoma cell lines to MEK inhibition.

## DISCUSSION

To identify new candidate avenues for therapeutic intervention for osteosarcoma we performed a kinase inhibitor screen in two human osteosarcoma cell lines. Our screen confirms previously reported findings (e.g. PI3K-AKT-mTOR inhibition), thereby validating our screen. It also identifies new drugs in the context of osteosarcoma that are in the clinic for other malignancies and hence may be candidates for repurposing.

PI3K-Akt-mTOR pathway is a network that controls many cellular processes such as cell proliferation, survival, metabolism and genomic integrity [27]. It has been shown that osteosarcoma strongly depends on this pathway for cell survival and proliferation and pathway inhibition triggers cell death [13, 28]. The expression of mTOR is correlated with event-free survival and cancer progression in osteosarcoma [29]. Our screen confirms mTOR signaling as a potential target to treat osteosarcoma.

The main characteristic of tumor cells is uncontrolled cell proliferation and cell cycle regulators are key players in cancer growth. Our screen identifies several inhibitors targeting this hallmark of cancer, including inhibitors of cyclin-dependent kinases and spindle checkpoints. Cyclin-dependent kinases 2,4 and 6 are altered in 80-90% of tumors[30]. In osteosarcoma, the Rb/p16/CDK4 axis is often deregulated with mutations or deletions in these genes [31, 32]. Aurora and polo-like kinases are critical regulators of the mitotic spindle and have been implicated in various cancers [33]. Several studies have shown that inhibition of Aurora kinases leads to cell death in osteosarcoma [34, 35]. The Aurora kinase A inhibitor Alisertib (not present in our library) is undergoing testing in a phase II clinical trial of refractory solid tumors (NCT01154816). Inhibition of polo like kinase (Plk) 1 causes growth inhibition in various cancers [5, 36]. In osteosarcoma, Plk1 show higher expression in tumor samples compared to normal tissue, and its inhibition with NMS-P397 (not present in our library) leads to growth arrest and apoptosis [22].

The Ras-Raf-MEK-ERK mitogen activated protein kinase cascade is known to be involved in cell proliferation, apoptosis, differentiation and development. It integrates signals from cell surface receptors to activate ERK, which in turn enters the nucleus and activates transcription factors such as c-Myc, c-Fos, Ets, and Elk-1 [37]. This pathway is often deregulated in tumors due to mutations or overexpression of upstream signaling components. *B-Raf* and *Ras* are frequently mutated in melanoma, colorectal cancer, ovarian cancer, lung cancer and pancreatic cancer among others [38, 39]. In osteosarcoma, ERK pathway activity was reported to occur in 67% of the cases analyzed, and mutations in *B-Raf* were only found in 13% of the cohort [11]. We identify three MEK inhibitors in the osteosarcoma cell viability screen: Trametinib is a selective allosteric inhibitor of MEK1/2 designed to treat tumors with overactive MEK-ERK pathway, which is found in tumors with *B-Raf* mutations [40]. It was approved for melanoma, and it has also been tested in patients with pancreatic cancer, colorectal cancer and other solid tumors with *B-Raf* mutations [41]. AZD8330 and TAK-733 are two selective allosteric MEK1/2 inhibitors [42, 43]. TAK-733 has shown good antitumor activity in melanoma cells [44] as well as in human lung cancer [45].

Our findings imply that MEK1/2 inhibition is a candidate approach to treat osteosarcomas harboring high ERK activity. Strikingly, while ERK phosphorylation status predicts sensitivity to MEK inhibition, mutation analysis of upstream components of this pathway does not identify candidate predictive mutations. Hence, ERK phosphorylation in tumor tissue as identified by immunohistochemistry may be a more accurate biomarker predicting sensitivity to MEK1/2 inhibitors than genomic analyses. We have not identified an alternative pathway selectively driving viability/growth of cell lines that are resistant to MEK inhibition. An enriched set of genes in the lines points to differential activation of the AKT pathway but based on AKT and mTOR phosphorylation status this pathway is active in all lines and, in agreement, all cell lines are similarly sensitive to AKT-mTOR inhibition. Interestingly, this indicates that three independent cell lines showing strong activity of MEK as well as AKT depend on the activity of both pathways. I.e., inhibition of either pathway is sufficient to cause loss of viability rather than these pathways compensating for each other.

To our knowledge, we are the first to describe the efficacy of MEK inhibition in osteosarcoma cells with high ERK phosphorylation. Recently, a Phase I clinical trial (NCT02124772) started enrolling patients with solid tumors, including osteosarcoma, to study the efficacy of trametinib in combination with dabrafenib. In this setting, such association between ERK phosphorylation status and response to trametinib may be investigated.

## MATERIALS AND METHODS

### Reagents and antibodies

The kinase inhibitor library (L1200), Trametinib, AZD8330 and TAK-733 inhibitors were purchased from SelleckChem (Huissen, Netherlands). The ERK (9102), phospho(44/42)-ERK (137F5), phospho(Ser2448)-mTOR (D9C2), phospho(Ser473)-AKT (#9271) and AKT (#9272) antibodies were from Cell Signaling (Bioké, Leiden, Netherlands). The antibody against tubulin (T-9026) was from Sigma Aldrich (Zwijndrecht, The Netherlands).

### Cell culture

Human osteosarcoma cell lines MOS, U2OS, 143B, ZK58, KPD and Saos-2 were previously described[46, 47]. Cells were grown in RPMI1640 medium supplemented with 10% fetal bovine serum and 25 U/mL penicillin and 25 μg/mL of penicillin-streptomycin. All cells were cultured in a humidified incubator at 37°C with 5% CO_2_.

### Immunoblotting

Cells were lysed with SDS protein buffer (125mM Tris/HCl pH 6.8, 20% glycerol, 4% SDS and 0.2% bromophenol blue). Proteins were resolved by SDS-PAGE and transferred to polyvinylidine difluoride membrane. Membranes were blocked in 5% BSA-TBST (TRIS-0.05% Tween20), followed by overnight incubation with primary antibodies and 45 minutes incubation with HRP-conjugated secondary antibodies. Chemiluminescence was detected with a Typhoon 9400 imager (GE Healthcare).

### Cell viability and caspase3/7 activity

Cells were processed using the ATPlite 1Step kit (Perkin Elmer) according to the manufacturer's instructions, followed by luminescence measurement on a plate reader. Caspase 3/7 activity was assessed with Caspase-Glo® 3/7 from Promega (Leiden, The Netherlands) according to manufacturer's protocol, and luminescence measurement on a plate reader.

### 3D culture assay

MOS and U2OS cells were cultured in 384-well plates (Greiner μclear) in a hydrogel containing Matrigel (Beckton Dickinson) and collagen I, supporting invasive growth of both cell lines. Cells in culture were trypsinized and directly added to the cooled gel solution. Using a robotic liquid handler (CyBio Selma 96/60), 14.5μL of gel-cell suspension was transferred to each well of a 384-well plate (2000 cells/well). After polymerization for 30 minutes at 37°C in an atmosphere of 5% CO_2_, growth medium was added on top of the gel. After 24 hours, the cells were exposed to the compounds in quadruplicate for a period of 72 hours. For measuring cell viability in 3D, ATPlite was used as indicated by the manufacturer and luminescence was measured using a FluoroStar plate reader. Percentage viability was thereafter calculated by normalization of all conditions to DMSO. Results are presented as means ± SD. Images of 3D cultures were taken with a BD Pathway 855 (BD Biosciences).

### Pathway analysis

We used a previously published dataset of mRNA expression of 19 osteosarcoma cell lines [Namlos et al PLoSOne 7, e48086, 2012] and performed a LIMMA analysis [48] of sensitive (MOS, U2OS and 143b) versus resistant (KPD, ZK58 and Saos-2) cell lines [26]. We then ran a pre-ranked gene set enrichment analysis [49] using MSigDb v5.0 BioCarta (http://www.biocarta.com) signatures on the Benjamini and Hochberg False Discovery Rate corrected p-values obtained from LIMMA. Statistically significant signatures were defined as signatures with FDR<0.25.

### Statistical analysis

Dose response curve fitting and statistical analyses were performed with GraphPad Prism 5.0 (GraphPad Software, La Jolla, CA).
